# Simultaneous Acquisition of Multicolor Information From Neural Circuits in Resin-Embedded Samples

**DOI:** 10.3389/fnins.2018.00885

**Published:** 2018-11-30

**Authors:** Miao Ren, Jiaojiao Tian, Peilin Zhao, Jialiang Luo, Zhao Feng, Hui Gong, Xiangning Li

**Affiliations:** ^1^Britton Chance Center for Biomedical Photonics, Wuhan National Laboratory for Optoelectronics, Huazhong University of Science and Technology, Wuhan, China; ^2^MoE Key Laboratory for Biomedical Photonics, Collaborative Innovation Center for Biomedical Engineering, School of Engineering Sciences, Huazhong University of Science and Technology, Wuhan, China; ^3^HUST-Suzhou Institute for Brainsmatics, Suzhou, China

**Keywords:** resin embedding, red fluorescent protein, preservation of fluorescence, single-neuron resolution, whole brain

## Abstract

Resin embedding has been widely used for precise imaging of fluorescently labeled biological samples with optical and electron microscopy. The low preservation rate of fluorescence, especially for red fluorescent proteins, has limited the application of resin embedding in multifluorescent protein-labeled samples. Here, we optimized the embedding method to retain the intensity of multiple fluorescent proteins during resin embedding. By reducing the polymerization temperature from 50 to 35°C and adding a fluorescent protein protection reagent during the embedding process, we successfully increased the fluorescence preservation rate by nearly twofold for red fluorescent proteins, including tdTomato, mCherry, and DsRed. Meanwhile, the background fluorescence decreased significantly in the optimized embedding method. This method is suitable not only for red fluorescent protein-labeled samples but also for blue (BFP) and green fluorescent protein (GFP)-labeled samples. We embedded brains labeled with BFP, DsRed, and GFP via AAV and rabies virus and acquired the distribution of input neurons to different cortical areas. With GFP/tdTomato double-labeled samples in resin, we obtained the cholinergic projectome of the pedunculopontine tegmental nucleus (PPTg) and the distribution of cholinergic neurons at single-neuron resolution in the whole brain simultaneously. Input cholinergic terminals from the PPTg were found to innervate the cholinergic soma and fiber in the neocortex, basal forebrain and brainstem, indicating that local cholinergic neurons received long-range cholinergic modulation from the midbrain. Our optimized method is useful for embedding multicolor fluorescent protein-labeled samples to acquire multidimensional structural information on neural circuits at single-neuron resolution in the whole brain.

## Introduction

Resinis widely used to produce ultrathin sections for embedding optical imaging and electron microscopy to acquire detailed structural information on biotissue (Newman and Hobot, 1999; Chen et al., 2008; Gong et al., 2016; Quan et al., 2016). Combining this method with transgenic techniques and viral tracing, we can label samples with fluorescent proteins and acquire specific structural information with high resolution in large samples such as the whole brain (Gong et al., 2016). The fluorescence intensity of green fluorescent protein (GFP) embedded in resin can be recovered by adjusting the pH value (Watanabe et al., 2011; Yang et al., 2013; Xiong et al., 2014). However, the fluorescence intensity of red fluorescent protein (RFP) is difficult to preserve in resin, which limits the application of resin-embedded samples in multicolor fluorescence signal acquisition. Although specific RFPs such as PHuji, which is pH sensitive, can be used to achieve good fluorescence preservation during resin embedding (Guo et al., 2017), popular RFPs such as mCherry, tdTomato, and DsRed lose most of their fluorescence intensity after resin embedding due to their insensitivity to pH. Meanwhile, the increased background fluorescence in resin-embedded samples can mask weak signals, which compromises the reconstruction of the full morphology of single neurons. With the development of transgenic techniques, we can label specific structures with different fluorescent proteins in a single sample. To obtain information on different structures simultaneously with precise imaging (Livet et al., 2007; Pan et al., 2011; Patterson et al., 2011), the development of resin-embedding methods compatible with multicolor fluorescent protein labeling is urgently needed.

In the present study, we modified the glycol methacrylate (GMA) embedding method by adding a fluorescent protein protection reagent and reducing the polymerization temperature. The optimized method increased the RFP fluorescence preservation rate nearly twofold. Meanwhile, this method simultaneously resulted in reduced autofluorescence. Using GFP- and tdTomato-labeled samples in resin, we simultaneously acquired the cholinergic projectome of the PPTg specifically labeled with GFP and the distribution of cholinergic neurons in the whole brain specifically labeled with tdTomato. These results demonstrated that the present embedding method is suitable for precise imaging of multicolor fluorescent protein-labeled biological samples.

## Materials and Methods

### Animals

Vasoactive intestinal peptide-positive (VIP)-Cre transgenic mice were obtained from Josh Huang’s lab (Cold Spring Harbor Laboratory, Cold Spring Harbor, NY, United States). VGAT-Cre mice, Ai14 mice and ChAT-IRES-Cre transgenic mice were obtained from the Jackson Laboratory.

To genetically label specific neurons, we crossed VIP-Cre mice and ChAT-ires-Cre mice with Ai14 reporter mice (VIP-Cre::Ai14 mice and ChAT-IRES-Cre::Ai14 mice), in which the tdTomato was expressed in a Cre-dependent manner. Mice were housed on a 12-h light/dark cycle with food and water *ad libitum*. Male mice older than 8 weeks were used in the study. All animal experiments followed procedures approved by the Animal Advisory Committee of Huazhong University of Science and Technology.

### Virus Injections

To label the morphology of specific neurons, we used Adeno-associated virus (AAV) and Cre-dependent mice. ChAT-IRES-Cre mouse was injected with 50 nL of AAV-CAG-Dio-mCherry into the mPFC to express mCherry in cholinergic neurons (BrainVTA Co., Ltd., Wuhan, China; serotype: 2/9, titer approximately 6 × 10^12^ genome copies per milliliter). Then, 50 nL of AAV-CAG-DIO-GFP was injected into the PPTg of the ChAT-IRES-Cre::Ai14 hybrid mice. The injections were conducted using a syringe pump (Item: 53311, Quintessential stereotaxic injector, Stoelting, United States) connected to a glass micropipette with a tip diameter of 10–15 mm. The glass micropipette was held for an extra 10 min after the completion of the injection and then slowly retracted. After surgery, the incisions were stitched, and the animals were returned to their home cages for recovery. After 21 days of recovery, the mice were perfused. To label the input neuron of the motor cortex, we used AAV helper and Glycoprotein-Deleted Rabies Viruses (RV, BrainVTA Co., Ltd., Wuhan, China.). First, AAV helper virus was mixed together (AAV-DIO-TVA-BFP and AAV-DIO-RG) in a ratio of 1:1. Then, 150 nL of mixed AAV was injected in the primary motor cortex (M1) of the left and right hemisphere in VGAT-Cre transgenic mice. Three weeks later, 300 nL of RV-ΔG-EnVA-EGFP and RV-ΔG-EnVA-DsRed was injected into the corresponding sites of the left and right hemisphere, respectively. Mice were perfused 7–10 days later.

Then, the brains were embedded using the following procedure.

### Resin Embedding

Embedding procedures were performed on 100-μm brain slices, which were dehydrated in a graded ethanol series (50, 70, and 95% ethanol, changing from one concentration to the next every 5 min at 4°C). After dehydration, the brains were immersed in a graded GMA series (Ted Pella, United States), including 0.2% Sudan black B (SBB)(Sun et al., 2011) (70, 85, and 100% GMA for 15 min each and 100% GMA overnight at 4°C). For whole-brain embedding, brains were dehydrated in a graded ethanol series every 1 h at 4°C and immersed in a graded GMA series for 2 h each and 100% GMA overnight at 4°C. Subsequently, the samples were impregnated in a prepolymerization GMA solution for 3 days at 4°C and embedded in a vacuum oven at 35°C for 24 h. The 100% GMA solution comprised 52.5 g of A solution, 2.8 g of deionized water, 44.1 g of B solution, 0.2 g of SBB, and 0.8 g of ABVN as an initiator. The 70 and 85% GMA solutions (wt/wt) were prepared from 95% ethanol and 100% GMA.

### Imaging

A commercial confocal microscope (Zeiss 710) was used to image the 100-μm brain sections. A series of optic slices was obtained, and projected images were generated. BFP was excited using a 415-nm laser, GFP was excited using a 488-nm laser, tdTomato was excited using a 514-nm laser, and mCherry/DsRed was excited using a 561-nm laser. We performed whole-brain data acquisition using BPS (Gong et al., 2016). Briefly, we used two-channel structured-illumination microscopy to image GFP and RFP simultaneously at a voxel resolution of 0.32 μm × 0.32 μm × 2 μm.

### Fluorescence Intensity Quantitative Analysis

Fluorescence intensity of the neurons in images was read out and analyzed using ImageJ software. First, we use the oval-selection tool to select the somas of neurons. Then, by using the histogram tool, we measured the mean fluorescence intensity of the oval area. The normalized fluorescence intensity was obtained by normalizing the mean fluorescence intensity by 255. We suppose that the mean fluorescence intensity of a neuron before treatment is I, and the mean fluorescence intensity of the neuron changes to I_o_ after treatment. Calculated The preservation ratio of the signal was calculated as I/I_o_ × 100%. The rising ratio of the background was (I-I_o_ )/I_o_ × 100%. We selected multiple neurons from three independent samples to determine the fluorescence intensity.

### Statistics

All statistical graphs were generated using GraphPad Prism 6.01. The two-tailed student’s *t*-test and one-way ANOVA followed by Tukey’s *post hoc* tests were also performed using GraphPad Prism 6.01 and SPSS (IBM SPSS Statistics 23). The confidence level was set to 0.05 (*P*-value), and all results are presented as the means ± SD.

## Results

### Increasing the Fluorescence Preservation Rate During the Resin-Embedding Process

Compared with other resins such as LR white, GMA has better penetration ability for biotissue and better fluorescence preservation capacity for GFP signals (Yang et al., 2013). However, the fluorescence preservation of RFP is low; for example, the intensity of tdTomato decreases 55.4 ± 8% after resin embedding. Thus, RFP-labeled signals are difficult to distinguish, especially for weak signals in small structures such as the terminals of the dendrites and thin axons.

Throughout the entire resin-embedding process, dehydration and infiltration are major steps. Reagents such as ethyl alcohol decrease the intensity of fluorescent proteins during these steps (Mao et al., 2011; Yang et al., 2013). Here, to increase the fluorescence preservation rate of RFP embedded in GMA, we added dithiothreitol (DTT) as a fluorescent protective agent in the dehydration and infiltration steps, which has been used to preserve GFP fluorescence in plant tissues (Baskin et al., 1992).

**FIGURE 1:**
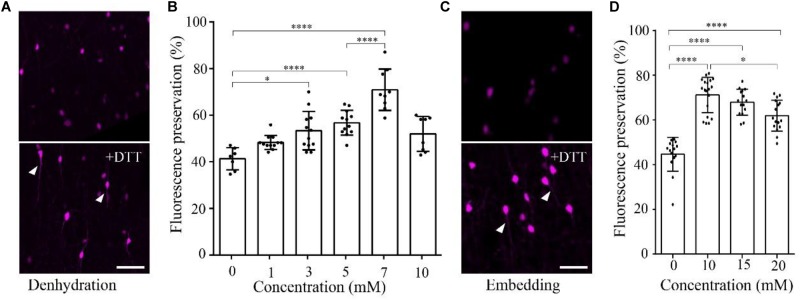
DTT increases the distribution fluorescence intensity of tdTomato during dehydration and embedding. **(A)** tdTomato-labeled VIP neurons in slices after dehydration without and with DTT (7 mM) (scale bar: 20 μm). **(B)** Fluorescent protein preservation ratios of neurons in dehydration agents containing different concentration of DTT (*n* = 7, 12, 12, 12, 9, and 8, respectively). Error bars represent SD. One-way ANOVA followed by Tukey’s *post hoc* tests (^∗^*p* < 0.05, ^∗∗∗∗^*p* < 0.001). **(C)** tdTomato-labeled VIP neurons in slices after being embedded without and with DTT (10 mM) (scale bar: 20 μm). **(D)** Fluorescent protein preservation ratios after embedding with different concentrations of DTT in the resin (*n* = 15, 19, 15, and 16, respectively). Error bars represent SD. One-way ANOVA followed by Tukey’s *post hoc* tests (^∗^*p* < 0.05, ^∗∗∗∗^*p* < 0.001).

To demonstrate the effect of DTT on the fluorescence preservation rate of RFP, we first added different concentrations of DTT to the dehydration agents and resin, and compared the fluorescence preservation of tdTomato. In transgenic mice (VIP-Cre mice crossed with Ai14 mice), VIP neurons expressed tdTomato in the soma and neurites. Following postfixation with 4% paraformaldehyde (PFA) for 24 h, the brain was cut into 100-μm-thick slices. Before and after embedding in resin with different concentrations of DTT, the brain slices were imaged via confocal microscopy, and the fluorescence intensity was analyzed with the ImageJ software. As shown in Figure 1, the fluorescence preservation rate increased with the concentration of DTT in the dehydration agents and infiltration resin. After dehydration, the fluorescence preservation rate was 41.3 ± 4.7% without DTT but increased to 70.9 ± 8.9% with 7 mM DTT in the dehydrating agents. However, the fluorescence preservation rate decreased to 51.9 ± 7.5% with 10 mM DTT (Figures 1A,B and Supplementary Table 1). Thus, we selected 7 mM as the optimal DTT concentration in the dehydration agents.

During the resin-infiltration step, the fluorescence preservation rate increased with the concentration of DTT in the resin and reached the highest value 71.1 ± 8.0% with 10 mM DTT in the resin. The somatic signals were brighter, and near-somatic fibers were observed, as indicated in Figure 1 (Supplementary Table 2). With more DTT added during resin infiltration, the fluorescence intensity decreased to 61.9 ± 6.9% at 20 mM DTT in the resin. These results indicate that different DTT concentrations at different steps, namely, 7 mM in the dehydrating agents and 10 mM during resin infiltration, yielded optimal fluorescence preservation.

### Reducing the Polymerization Temperature Increases the Signal-to-Noise Ratio

As previously reported, the fluorescence intensity of fluorescent protein decreases in higher-temperature environments (Zhang et al., 2009). To preserve fluorescence, we sought to decrease the polymerization temperature of GMA when embedding the fluorescent protein-labeled samples.

**FIGURE 2:**
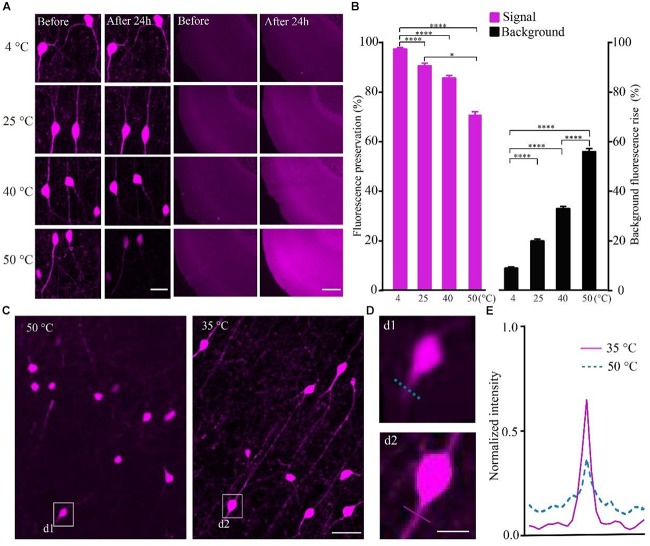
Increasing the fluorescent intensity of tdTomato and decreasing the background fluorescence with lower polarization temperatures. **(A)** tdTomato fluorescence and background fluorescence at different temperatures (scale bar in left column: 20 μm; scale bar in right column: 500 μm). **(B)** Fluorescent protein preservation ratio and the background fluorescence rising rate at different temperature (each column filled with magenta *n* = 8, each column filled with black *n* = 3). Error bars represent SD. One-way ANOVA followed by Tukey’s *post hoc* tests (^∗^*p* < 0.05, ^∗∗∗∗^*p* < 0.001). **(C)** Fluorescence brightness of the tdTomato fluorescence (scale bar: 20 μm). **(D)** Enlarged images from C showing the fibers around the soma after embedding at different conditions (scale bar: 10 μm). **(E)** Fluorescence intensity plots for the lines labeled in (d1 and d2).

To quantify the effect of temperature on the RFP, we calculated the intensity of tdTomato in VIP-Cre::Ai14 mice brain slices imaged via confocal microscopy after being kept at different temperatures. The results show that, after slice were kept in PBS for 24 h, the higher-temperature group showed not only reduced fluorescent intensity but also increased tissue autofluorescence (Figure 2A). In the 4, 40, and 50°C groups, the fluorescence preservation rate was 97.0 ± 1.4, 85.2 ± 2.7, and 70.4 ± 3.4%, while the autofluorescence increased by 8.8 ± 0.9, 32.7 ± 1.4, and 55.5 ± 2.1% (Figure 2B and Supplementary Table 3), respectively. The results indicated that higher polymerization temperatures effectively decrease the signal-to-noise ratio of red fluorescent samples, especially for temperatures above 40°C. However, the polymerization temperature of GMA resin was set as 48°C in previous works (Gong et al., 2016). To decrease the polymerization temperature, we replaced the initiator azodiisobutyronitrile (AIBN) with 2, 2′-Azobis-(2,4-dimethylvaleronitrile) (ABVN), which can work at lower temperatures. We then tested different amounts of ABVN in the resin at different temperatures for polymerization. The best ABVN amount was 0.8 g in 100 g resin, and the lowest polymerization temperature was 35°C.

To test the effect of the lower polymerization temperature on the preservation rate of RFP, we embedded the tdTomato-labeled brain slices at 35°C using ABVN. After embedding, the fluorescence preservation rate of RFP was compared with that in the previous method at 48°C using AIBN. Although the VIP neurons mainly project in local areas, fibers with multiple branches were visible after embedding at 35°C, and lower background was presented compared with the traditional embedding method at 50°C (Figure 2C). The signal increased and the background fluorescence decreased (Figures 2D,E and Supplementary Table 4) in the ABVN/GMA-embedded samples.

### Fluorescence Preservation of RFP/GFP/BFP in Optimized Resin Embedding

To obtain higher fluorescence preservation rates of RFP embedded in resin, we added the fluorescence protective agent and reduced the temperature for the entire procedure. Using the optimized method, we embedded tdTomato-labeled brain slices and quantified the fluorescence preservation rate. The 100-μm-thick brain slices were dehydrated in a graded series of ethanol solutions with DTT for 5 min and subsequently infiltrated in a graded series of resin solutions with DTT for 15 min, and were then placed in the prepolymer of GMA. After 6 h, the brain slices were embedded in an oven at 35°C.

**FIGURE 3:**
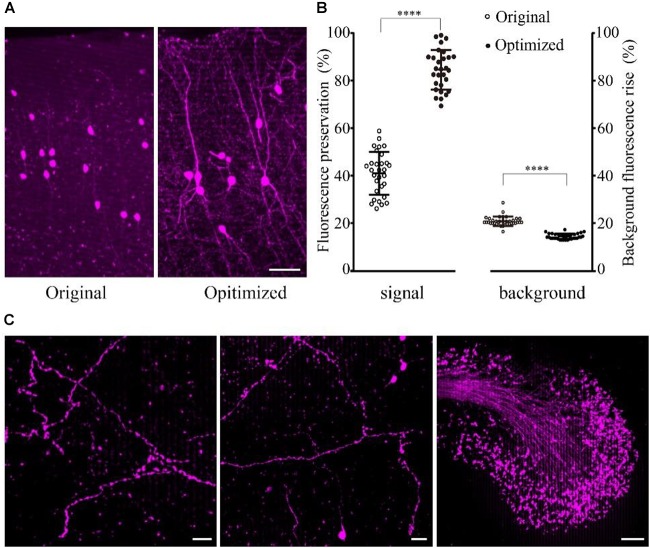
Preservation of fluorescence after resin embedding. **(A)** The VIP::tdTomato samples in normal and optimized GMA resin (scale bar: 30 μm). **(B)** Fluorescent protein preservation ratio and the background fluorescence rising rate after embedding (*n* = 30, 29, 36, 36 from left to right). Error bars represent SD. One-way ANOVA followed by Tukey’s *post hoc* tests (^∗∗∗∗^*p* < 0.001). **(C)** Fine structures were clearly detected with the optimized embedding method (scale bar in the first two columns: 20 μm; Scale bar in the last column: 50 μm).

The results showed that the fine structure of the tdTomato-labeled neurons was visible in the samples embedded using the optimized method (Figure 3A). The fluorescence preservation rate of RFP (tdTomato) increased from 41.0 ± 9.0 to 86.6 ± 8.3%, while the background fluorescence was effectively decreased from 20.8 ± 2.0 to 14.4 ± 1.1% compared with the original method without DTT and 48°C as the polymerization temperature (Figure 3B and Supplementary Table 5). In the samples, tiny axons and buttons were clearly detected in some brain regions (Figure 3C). These results demonstrated that our new embedding method is suitable for embedding RFP-labeled samples.

**FIGURE 4:**
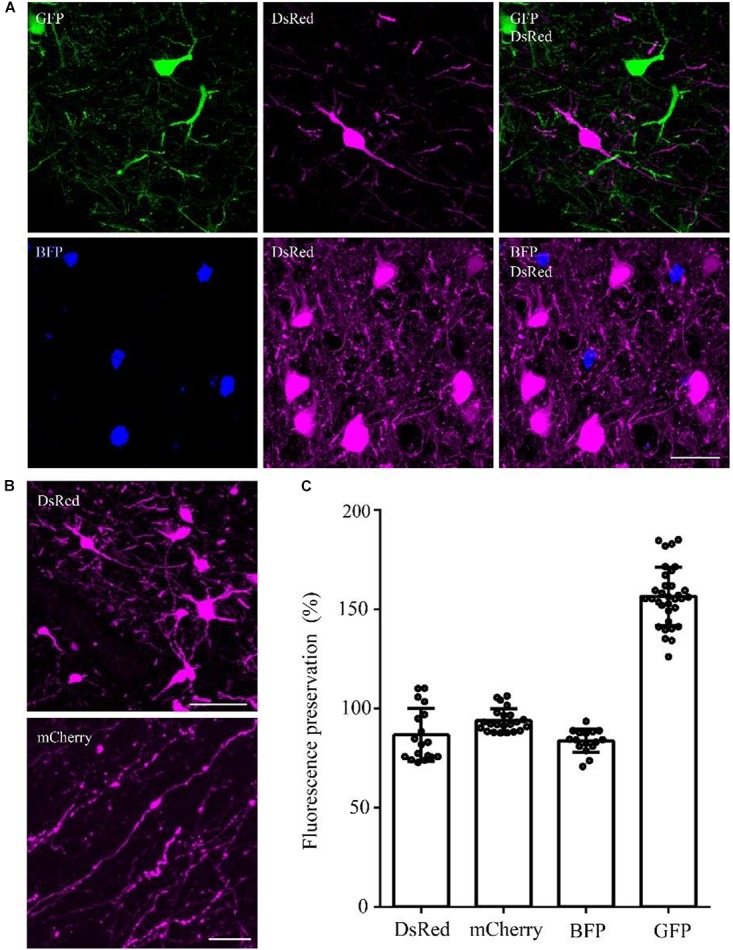
DsRed/mCherry/GFP/BFP can be well preserved using the optimized GMA embedding method. **(A)** Using viral tracing (AAV+RV), we labeled the GABAergic input neurons of M1 in VGAT-Cre mice. GFP represented the input neurons of M1 in the right hemisphere, DsRed represented the input neurons of M1 in the left hemisphere, and BFP showed the start neurons in M1. The fluorescence of each protein was clearly detected following the optimized embedding method (scale bar: 20 μm). **(B)** DsRed and mCherry signals after embedding (scale bar in top column: 20 μm; scale bar in bottom column: 5 μm). **(C)** Fluorescent proteins preservation ratios after optimized embedding (*n* = 18, 22, 16, 33 from left to right). Error bars represent SD.

To confirm whether the optimized method can be used for multiple fluorescent proteins, we tested this method on DsRed/mCherry/GFP/BFP-labeled samples. After being embedded in resin with the optimized method, the DsRed signal was well preserved in viral-tracing samples (Figure 4A). The cell body and thin wire-like axons labeled by mCherry showed no notable differences in transgenic mice and viral-tracing samples before and after being embedded in resin using the optimized method (Figure 4A).

In addition, the fluorescence preservation rate of DsRed was 86.8 ± 13.4%, mCherry was 94 ± 5.8%, BFP was 83.7 ± 5.9%, and GFP was 156.6 ± 14.7% (Figure 4C and Supplementary Table 6). We labeled input neurons of the motor cortex in the right and left hemisphere with GFP and DsRed rabies virus simultaneously. Then, we tested the embedding methods in these samples labeled with blue fluorescent protein (BFP), DsRed and GFP together. Here, BFP labeled the RV-infected neurons in the motor cortex, DsRed labeled the input neurons to the left motor cortex, and GFP labeled the input neurons to the right motor cortex. As shown in Figure 4C, DsRed, GFP and BFP signals were all well preserved. The merged images of the BFP and DsRed signals showed the start neurons of the motor cortex, and localization information indicated that more than half of the RV-infected neurons were start neurons. The GFP and DsRed signals showed the input neurons projecting to GABAergic neurons of the left and right hemispheres, respectively, which were different in the same brain region (Figure 4C). These results showed that the optimized GMA embedding method is suitable for BFP/GFP/tdTomato/DsRed and mCherry labeled samples.

### Simultaneous Acquisition of GFP/tdTomato-Labeled Information in Embedded Whole Brain

**FIGURE 5:**
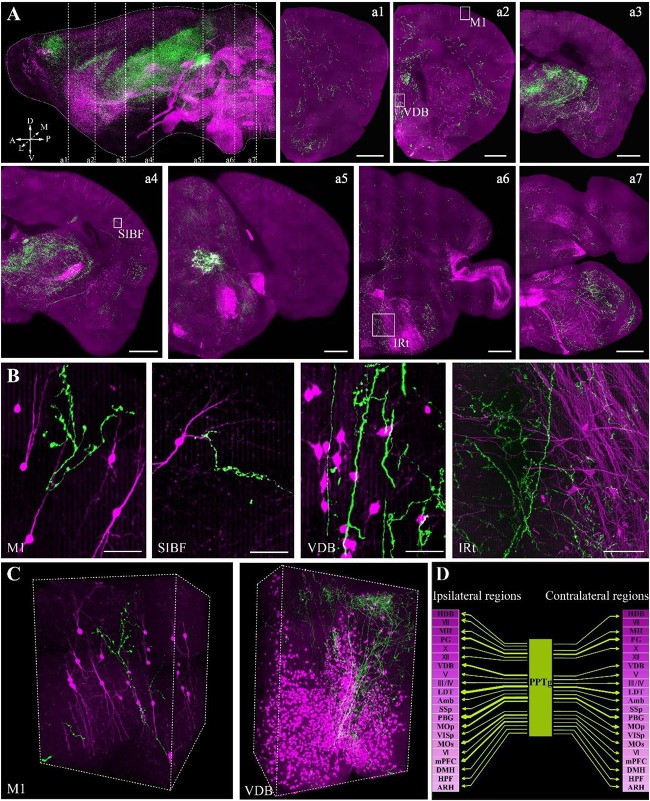
Whole-brain imaging of dual color-labeled neurons in a ChAT-IRES-Cre::Ai14 mouse brain. **(A)** Sagittal reconstruction of the maximum intensity projection and continuous coronal sections in the ChAT-IRES-Cre::Ai14 mouse brain. Projection thickness was 100 μm. Maximum-intensity projections were reconstructed at a resolution of 2 μm × 2 μm × 2 μm. Red signals indicate tdTomato-positive cholinergic neurons, while green signals are GFP-labeled cholinergic fibers from the PPTg. A, anterior; P, posterior; D, dorsal; V, ventral; M, medial; L, lateral (scale bar: 500 μm). **(B)** The enlarged details of A (scale bar: 50 μm). **(C)** 3D reconstructions of the GFP and tdTomato signals in M1 and VDB, respectively. **(D)** The projections of the cholinergic PPTg to other cholinergic neuron-enriched nuclei. Crimson represents a dense distribution of cholinergic neurons, and lighter colors signify fewer cholinergic neurons in the nucleus. Arrow thickness represents the density of cholinergic projection from the PPTg.

To confirm whether the optimized embedding method was suitable for precise imaging in the whole brain, we embedded brains labeled with two fluorescent proteins, in which tdTomato labeled all cholinergic neurons in the whole brain and GFP specifically labeled the cholinergic projections of the PPTg, a brainstem nucleus enriched with cholinergic neurons. Using our precise whole-brain imaging system (Gong et al., 2016; Quan et al., 2016), GFP and tdTomato signals were obtained simultaneously at single-neuron resolution in the whole brain (Figure 5A). The continuous 3D dataset allowed us to locate each labeled neuron in all regions containing cholinergic neurons in the whole brain, which can be used to quantify the exact number in specific region and reconstruct the projectome.

The locations of cholinergic neurons in the whole brain were obtained using tdTomato, as had been reported in our previous work with Chat-IRES-Cre::Ai47 mice, which, containing three GFP genes, yielded much higher fluorescence intensity than a single GFP cassette (Li et al., 2018). In present study, with tdTomato labeling, the soma of cholinergic neurons and the dendrites around the soma were visible in the cortex (Figure 5B), and the long projecting cholinergic fibers showing tdTomato were also visible in the brainstem (Figures 5A,B). The cholinergic terminals from the PPTg innervated multiple brain regions, including the motor cortex, sensory cortex, basal forebrain, thalamus, midbrain and hindbrain (Figure 5C). Moreover, we found that the PPTg can innervate many cholinergic neuron-enriched brain regions, such as the basal forebrain and some hindbrain nuclei. The cholinergic projections from the PPTg are rich in the VDB and LDT but rare in other cholinergic neuron-enriched regions, including MH and V/VII/IV/X in the brainstem, indicating that the cholinergic PPTg has direct connections to local cholinergic neurons in other brain regions and prefers to modify specific local neurons in the basal forebrain and midbrain (Figure 5D). Our results demonstrated that our optimized embedding method is suitable for embedding multicolor fluorescent protein-labeled biological samples for precise whole-brain imaging.

## Discussion

In this study, we reported an optimized GMA-embedding method for multicolor fluorescent protein-labeled samples, which can retain over 95% of the fluorescence intensity of GFP, RFP (including tdTomato, DsRed, and mCherry) and BFP. This method can be used to acquire multi-structure information from BFP/GFP/RFP-labeled samples.

Dehydrating agents and organic reagents are commonly known to reduce the fluorescence intensity of fluorescent proteins. DTT has been used in sample preparation to protect biomolecules (Thompson and Wolniak, 2008; Bell et al., 2013). We quantitated the effect of including DTT during the dehydration and infiltration processes for resin embedding and determined the optimal DTT concentration that yielded the highest fluorescence preservation rate. Our results showed that when the right concentration of DTT was used, the fluorescence preservation rate can increase almost 30%. This method can also be used in other fluorescent sample preparations for fluorescence microscopy.

For resin polymerization, the polymerization rate is affected by the temperature and the polymerization initiator. The fluorescence will be reduced during the polymerization process in higher-temperature environments (Yang et al., 2013; Gang et al., 2017). A lower polymerization temperature is beneficial for retaining the fluorescence intensity. The polymerization temperature highly depends on the polymerization initiator. It has been discovered that polymerization accelerators together with peroxide initiators can also initiate the polymerization at lower temperature, such as the combination of N, N-dimethyl aniline (DMBA) and benzoyl peroxide (BPO) (Gerrits and Horobin, 2013). In our previous study, we used Sudan black B (SBB) as the background inhibitor, which is reactive with BPO. So, we replaced the BPO with an azo initiator (Gong et al., 2016). Here, we used ABVN instead of AIBN to reduce the polymerization temperature from 50 to 35°C, which significantly increased the fluorescence preservation rate and signal-to-noise ratio. We tested different amounts of ABVN in the resin at different temperatures for polymerization. The best ABVN amount was 0.8 g in 100 g resin, and the lowest polymerization temperature was 35°C. ABVN can work at 4°C with special conditions, such as UV irradiation. But, for the limitation of UV penetration ability, UV polymerization can only be applied to small volumes tissues (Newman and Hobot, 1999). UV polymerization with ABVN at 4°C was not suitable for embedding the whole mouse brain.

This optimized method is also suitable for other fluorescent proteins such as GFP, mCherry, DsRed and BFP. The average fluorescence preservation rate of mCherry was 97%, while the highest fluorescence preservation rate was 106%. Similarly, the GFP signals average preservation rate was 156.6%. Dehydration and polymerization in the process of embedding can cause tissue shrinking and increased fluorescence intensity of some signals. Just like the statistic results for DsRed, mCherry and GFP signals as shown in Figure 4B. To further test whether the optimized method is suitable for embedding multi-fluorescent protein-labeled samples for precise whole-brain imaging, we also embedded whole brains that had been labeled with multiple fluorescent proteins. One set of samples was simultaneously labeled with BFP, DsRed and GFP, and another set of samples was labeled with tdTomato and GFP, in which tdTomato labeled all cholinergic neurons in whole brain and GFP labeled the cholinergic projection of the PPTg. By performing precise whole-brain imaging (Gong et al., 2016), we were able to clearly visualize the distribution of cholinergic neuron somas in all brain regions and the axons of the PPTg within the entire brain. Our results are consistent with previous studies of axon projections in the PPTg. We also found some new projection targets of the PPTg, such as the motor cortex and sensory cortex, which have not been reported previously.

Our results demonstrated that the optimized GMA method is fully capable of embedding multicolor fluorescent protein-labeled samples with a high fluorescence preservation rate and is suitable for precise whole-brain imaging. Multicolor fluorescent protein labeling and resin embedding are both widely used in neurobiology. Briefly, this optimized resin embedding method can combine multicolor labeling and ultrathin sectioning together for high-resolution microscopic imaging to acquire multidimensional information on complex neural circuits within individual brain samples. Meanwhile, this method has the potential to embed samples for correlated fluorescence microscopy and electron microscopy studies, as well as other fluorescence microscopy studies.

## Author Contributions

XL and HG conceived and designed the study. MR and JT performed the experiments. PZ performed the virus injections. JL performed the data acquisition. MR analyzed the data. ZF performed the imaging processing. XL and MR wrote the paper.

## Conflict of Interest Statement

The authors declare that the research was conducted in the absence of any commercial or financial relationships that could be construed as a potential conflict of interest.
