# Supplementary material for: Simultaneous Acquisition of Multicolor Information From Neural Circuits in Resin-Embedded Samples
Source: Front Neurosci. 2018 Nov 30;12:885. doi: 10.3389/fnins.2018.00885 (PMC6284031; doi:10.3389/fnins.2018.00885)
Supplement: Supplementary file 1 [file Table_1.docx]

**Supplementary Tables**

**Tables 1-6 are the raw data of the quantitative graphs.**

Table 1. The raw data of fluorescent protein preservation ratios of neurons in dehydration agents containing different concentration of DTT as shown in Fig1B.

| x-axis( different concentration of DTT mM) | 0.00 | 1.00 | 3.00 | 5.00 | 7.00 | 10.00 |
| --- | --- | --- | --- | --- | --- | --- |
| y-axis(fluorescent protein preservation ratio %) | 45.88 | 45.29 | 47.65 | 59.41 | 62.94 | 58.82 |
| y-axis(fluorescent protein preservation ratio %) | 42.35 | 48.24 | 70.00 | 54.12 | 58.82 | 58.24 |
| y-axis(fluorescent protein preservation ratio %) | 35.88 | 44.12 | 54.71 | 58.24 | 68.82 | 58.24 |
| y-axis(fluorescent protein preservation ratio %) | 34.71 | 47.06 | 64.71 | 55.29 | 77.65 | 42.94 |
| y-axis(fluorescent protein preservation ratio %) | 40.00 | 50.59 | 53.53 | 51.76 | 79.41 | 45.29 |
| y-axis(fluorescent protein preservation ratio %) | 43.53 | 50.59 | 50.00 | 64.71 | 70.00 | 60.00 |
| y-axis(fluorescent protein preservation ratio %) | 47.06 | 48.24 | 44.12 | 52.94 | 68.24 | 44.12 |
| y-axis(fluorescent protein preservation ratio %) |  | 47.06 | 58.82 | 47.06 | 87.06 | 48.24 |
| y-axis(fluorescent protein preservation ratio %) |  | 55.88 | 47.06 | 52.94 | 65.29 |  |
| y-axis(fluorescent protein preservation ratio %) |  | 47.65 | 47.65 | 61.76 |  |  |
| y-axis(fluorescent protein preservation ratio %) |  | 47.06 | 58.24 | 58.82 |  |  |
| y-axis(fluorescent protein preservation ratio %) |  | 48.24 | 44.12 | 64.12 |  |  |

Table 2. The raw data of fluorescent protein preservation ratios of neurons after embedding with different concentrations of DTT in the resin as shown in Fig1D.

| x-axis( different concentration of DTT mM) | 0.00 | 10.00 | 15.00 | 20.00 |
| --- | --- | --- | --- | --- |
| y-axis(fluorescent protein preservation ratio %) | 41.18 | 73.73 | 68.63 | 68.63 |
| y-axis(fluorescent protein preservation ratio %) | 48.63 | 80.78 | 62.75 | 70.98 |
| y-axis(fluorescent protein preservation ratio %) | 50.98 | 70.98 | 74.12 | 66.67 |
| y-axis(fluorescent protein preservation ratio %) | 34.31 | 80.04 | 77.25 | 64.71 |
| y-axis(fluorescent protein preservation ratio %) | 50.20 | 78.43 | 68.63 | 70.20 |
| y-axis(fluorescent protein preservation ratio %) | 43.92 | 72.94 | 70.59 | 71.76 |
| y-axis(fluorescent protein preservation ratio %) | 48.24 | 76.86 | 72.94 | 67.45 |
| y-axis(fluorescent protein preservation ratio %) | 22.16 | 61.18 | 58.04 | 55.69 |
| y-axis(fluorescent protein preservation ratio %) | 49.41 | 58.43 | 64.71 | 52.16 |
| y-axis(fluorescent protein preservation ratio %) | 51.37 | 73.33 | 66.67 | 59.22 |
| y-axis(fluorescent protein preservation ratio %) | 47.06 | 74.12 | 72.55 | 56.86 |
| y-axis(fluorescent protein preservation ratio %) | 45.10 | 58.43 | 73.73 | 57.25 |
| y-axis(fluorescent protein preservation ratio %) | 46.27 | 59.02 | 66.67 | 59.61 |
| y-axis(fluorescent protein preservation ratio %) | 43.14 | 66.27 | 62.75 | 60.78 |
| y-axis(fluorescent protein preservation ratio %) | 47.28 | 77.65 | 58.59 | 58.82 |
| y-axis(fluorescent protein preservation ratio %) |  | 78.04 |  | 49.41 |
| y-axis(fluorescent protein preservation ratio %) |  | 76.47 |  |  |
| y-axis(fluorescent protein preservation ratio %) |  | 60.39 |  |  |
| y-axis(fluorescent protein preservation ratio %) |  | 74.73 |  |  |

Table 3．The raw data of fluorescent protein preservation ratio and the background fluorescence rising rate at different temperature as shown in Fig2B.

| x-axis( different temperature °C) | 4.00 | 25.00 | 40.00 | 50.00 |
| --- | --- | --- | --- | --- |
| y-axis(fluorescent protein preservation ratio %) | 98.68 | 90.39 | 86.39 | 63.75 |
| y-axis(fluorescent protein preservation ratio %) | 95.65 | 94.69 | 84.69 | 71.52 |
| y-axis(fluorescent protein preservation ratio %) | 96.60 | 88.82 | 81.82 | 68.86 |
| y-axis(fluorescent protein preservation ratio %) | 97.19 | 86.86 | 85.86 | 70.07 |
| y-axis(fluorescent protein preservation ratio %) | 98.52 | 87.82 | 80.82 | 72.07 |
| y-axis(fluorescent protein preservation ratio %) | 96.60 | 94.69 | 88.68 | 75.78 |
| y-axis(fluorescent protein preservation ratio %) | 97.52 | 88.82 | 87.48 | 71.49 |
| y-axis(fluorescent protein preservation ratio %) | 94.60 | 89.12 | 86.12 | 69.58 |
|  |  |  |  |  |
| x-axis( different temperature °C) | 4.00 | 25.00 | 40.00 | 50.00 |
| y-axis( background fluorescence rising rate %) | 8.57 | 20.57 | 33.45 | 57.39 |
| y-axis( background fluorescence rising rate %) | 8.04 | 19.96 | 31.07 | 56.00 |
| y-axis( background fluorescence rising rate %) | 9.80 | 17.99 | 33.43 | 53.26 |

Table 4．The raw data of fluorescence intensity plots for the lines labeled in (d1 d2) as shown in Fig2E.

| x-axis( different temperature °C) | 35.00 | 50.00 |
| --- | --- | --- |
| y-axis(normalized intensity) | 0.15 | 0.05 |
| y-axis(normalized intensity) | 0.12 | 0.04 |
| y-axis(normalized intensity) | 0.11 | 0.03 |
| y-axis(normalized intensity) | 0.12 | 0.04 |
| y-axis(normalized intensity) | 0.13 | 0.06 |
| y-axis(normalized intensity) | 0.14 | 0.05 |
| y-axis(normalized intensity) | 0.14 | 0.06 |
| y-axis(normalized intensity) | 0.14 | 0.05 |
| y-axis(normalized intensity) | 0.17 | 0.04 |
| y-axis(normalized intensity) | 0.19 | 0.07 |
| y-axis(normalized intensity) | 0.19 | 0.17 |
| y-axis(normalized intensity) | 0.23 | 0.39 |
| y-axis(normalized intensity) | 0.37 | 0.65 |
| y-axis(normalized intensity) | 0.25 | 0.31 |
| y-axis(normalized intensity) | 0.20 | 0.12 |
| y-axis(normalized intensity) | 0.16 | 0.06 |
| y-axis(normalized intensity) | 0.15 | 0.05 |
| y-axis(normalized intensity) | 0.17 | 0.06 |
| y-axis(normalized intensity) | 0.13 | 0.07 |
| y-axis(normalized intensity) | 0.11 | 0.06 |
| y-axis(normalized intensity) | 0.10 | 0.04 |
| y-axis(normalized intensity) | 0.11 | 0.03 |
| y-axis(normalized intensity) | 0.14 | 0.04 |
| y-axis(normalized intensity) | 0.13 | 0.06 |
| y-axis(normalized intensity) | 0.12 | 0.07 |

Table 5．The raw data of fluorescent protein preservation ratio and the background fluorescence rising rate after embedding as shown in Fig3B.

| x-axis | signal-original | signal-optimized |  | x-axis | background-original | background-optimized |
| --- | --- | --- | --- | --- | --- | --- |
| y-axis(fluorescent protein preservation ratio %) | 45.20 | 75.23 |  | y-axis( background fluorescence rising rate %) | 20.00 | 12.94 |
| y-axis(fluorescent protein preservation ratio %) | 49.00 | 72.32 |  | y-axis( background fluorescence rising rate %) | 20.39 | 13.33 |
| y-axis(fluorescent protein preservation ratio %) | 42.30 | 97.78 |  | y-axis( background fluorescence rising rate %) | 22.75 | 14.12 |
| y-axis(fluorescent protein preservation ratio %) | 27.78 | 85.32 |  | y-axis( background fluorescence rising rate %) | 19.61 | 13.33 |
| y-axis(fluorescent protein preservation ratio %) | 45.31 | 90.05 |  | y-axis( background fluorescence rising rate %) | 20.00 | 13.73 |
| y-axis(fluorescent protein preservation ratio %) | 36.57 | 84.85 |  | y-axis( background fluorescence rising rate %) | 20.78 | 14.12 |
| y-axis(fluorescent protein preservation ratio %) | 44.02 | 89.91 |  | y-axis( background fluorescence rising rate %) | 20.00 | 16.08 |
| y-axis(fluorescent protein preservation ratio %) | 40.00 | 82.50 |  | y-axis( background fluorescence rising rate %) | 20.78 | 17.25 |
| y-axis(fluorescent protein preservation ratio %) | 30.91 | 84.31 |  | y-axis( background fluorescence rising rate %) | 21.18 | 15.69 |
| y-axis(fluorescent protein preservation ratio %) | 42.51 | 74.01 |  | y-axis( background fluorescence rising rate %) | 21.96 | 15.69 |
| y-axis(fluorescent protein preservation ratio %) | 44.30 | 76.17 |  | y-axis( background fluorescence rising rate %) | 22.75 | 16.47 |
| y-axis(fluorescent protein preservation ratio %) | 28.07 | 78.06 |  | y-axis( background fluorescence rising rate %) | 20.39 | 15.69 |
| y-axis(fluorescent protein preservation ratio %) | 35.52 | 75.51 |  | y-axis( background fluorescence rising rate %) | 22.35 | 14.51 |
| y-axis(fluorescent protein preservation ratio %) | 52.58 | 72.59 |  | y-axis( background fluorescence rising rate %) | 21.96 | 13.73 |
| y-axis(fluorescent protein preservation ratio %) | 39.36 | 69.36 |  | y-axis( background fluorescence rising rate %) | 24.71 | 14.90 |
| y-axis(fluorescent protein preservation ratio %) | 55.59 | 89.59 |  | y-axis( background fluorescence rising rate %) | 20.78 | 14.12 |
| y-axis(fluorescent protein preservation ratio %) | 52.57 | 78.87 |  | y-axis( background fluorescence rising rate %) | 21.96 | 15.29 |
| y-axis(fluorescent protein preservation ratio %) | 58.80 | 85.09 |  | y-axis( background fluorescence rising rate %) | 22.75 | 14.12 |
| y-axis(fluorescent protein preservation ratio %) | 45.09 | 80.53 |  | y-axis( background fluorescence rising rate %) | 20.78 | 13.73 |
| y-axis(fluorescent protein preservation ratio %) | 50.52 | 89.76 |  | y-axis( background fluorescence rising rate %) | 18.82 | 13.73 |
| y-axis(fluorescent protein preservation ratio %) | 26.13 | 92.15 |  | y-axis( background fluorescence rising rate %) | 21.18 | 12.94 |
| y-axis(fluorescent protein preservation ratio %) | 29.76 | 89.20 |  | y-axis( background fluorescence rising rate %) | 19.61 | 13.73 |
| y-axis(fluorescent protein preservation ratio %) | 52.15 | 96.13 |  | y-axis( background fluorescence rising rate %) | 20.39 | 15.29 |
| y-axis(fluorescent protein preservation ratio %) | 40.20 | 92.85 |  | y-axis( background fluorescence rising rate %) | 19.22 | 14.51 |
| y-axis(fluorescent protein preservation ratio %) | 44.84 | 98.47 |  | y-axis( background fluorescence rising rate %) | 18.82 | 13.33 |
| y-axis(fluorescent protein preservation ratio %) | 28.44 | 82.32 |  | y-axis( background fluorescence rising rate %) | 20.39 | 14.90 |
| y-axis(fluorescent protein preservation ratio %) | 42.32 | 87.80 |  | y-axis( background fluorescence rising rate %) | 20.39 | 13.73 |
| y-axis(fluorescent protein preservation ratio %) | 37.80 | 82.56 |  | y-axis( background fluorescence rising rate %) | 20.00 | 13.73 |
| y-axis(fluorescent protein preservation ratio %) | 29.06 | 99.06 |  | y-axis( background fluorescence rising rate %) | 19.61 | 15.29 |
| y-axis(fluorescent protein preservation ratio %) | 33.50 |  |  | y-axis( background fluorescence rising rate %) | 20.39 | 16.47 |
|  |  |  |  | y-axis( background fluorescence rising rate %) | 20.00 | 14.90 |
|  |  |  |  | y-axis( background fluorescence rising rate %) | 20.78 | 14.51 |
|  |  |  |  | y-axis( background fluorescence rising rate %) | 19.22 | 15.29 |
|  |  |  |  | y-axis( background fluorescence rising rate %) | 16.47 | 12.94 |
|  |  |  |  | y-axis( background fluorescence rising rate %) | 20.00 | 12.94 |
|  |  |  |  | y-axis( background fluorescence rising rate %) | 28.63 | 12.94 |

Table 6. The raw data of fluorescent proteins preservation ratios after optimized embedding as shown in Fig4C.

| x-axis (fluorescent proteins) | DsRed | mCherry | BFP | GFP |
| --- | --- | --- | --- | --- |
| y-axis(fluorescent protein preservation ratio %) | 73.97 | 90.25 | 82.56 | 126.11 |
| y-axis(fluorescent protein preservation ratio %) | 110.23 | 91.41 | 70.82 | 134.29 |
| y-axis(fluorescent protein preservation ratio %) | 75.77 | 106.40 | 88.41 | 135.19 |
| y-axis(fluorescent protein preservation ratio %) | 75.81 | 91.96 | 84.23 | 139.72 |
| y-axis(fluorescent protein preservation ratio %) | 110.12 | 97.89 | 84.35 | 140.31 |
| y-axis(fluorescent protein preservation ratio %) | 81.93 | 96.72 | 88.96 | 141.19 |
| y-axis(fluorescent protein preservation ratio %) | 88.51 | 105.65 | 81.01 | 141.28 |
| y-axis(fluorescent protein preservation ratio %) | 103.53 | 93.25 | 78.84 | 143.82 |
| y-axis(fluorescent protein preservation ratio %) | 76.25 | 88.25 | 93.66 | 149.27 |
| y-axis(fluorescent protein preservation ratio %) | 105.83 | 87.72 | 84.66 | 150.83 |
| y-axis(fluorescent protein preservation ratio %) | 77.37 | 94.45 | 81.04 | 152.03 |
| y-axis(fluorescent protein preservation ratio %) | 72.85 | 101.60 | 73.77 | 152.87 |
| y-axis(fluorescent protein preservation ratio %) | 74.21 | 104.26 | 88.97 | 153.65 |
| y-axis(fluorescent protein preservation ratio %) | 95.02 | 92.01 | 87.81 | 155.20 |
| y-axis(fluorescent protein preservation ratio %) | 75.63 | 88.07 | 87.45 | 155.30 |
| y-axis(fluorescent protein preservation ratio %) | 97.02 | 88.90 | 83.15 | 155.41 |
| y-axis(fluorescent protein preservation ratio %) | 84.79 | 87.82 |  | 155.42 |
| y-axis(fluorescent protein preservation ratio %) | 83.04 | 93.26 |  | 156.19 |
| y-axis(fluorescent protein preservation ratio %) |  | 96.65 |  | 156.45 |
| y-axis(fluorescent protein preservation ratio %) |  | 88.51 |  | 157.12 |
| y-axis(fluorescent protein preservation ratio %) |  | 90.93 |  | 158.29 |
| y-axis(fluorescent protein preservation ratio %) |  | 92.14 |  | 159.52 |
| y-axis(fluorescent protein preservation ratio %) |  |  |  | 159.60 |
| y-axis(fluorescent protein preservation ratio %) |  |  |  | 161.89 |
| y-axis(fluorescent protein preservation ratio %) |  |  |  | 161.97 |
| y-axis(fluorescent protein preservation ratio %) |  |  |  | 167.30 |
| y-axis(fluorescent protein preservation ratio %) |  |  |  | 169.52 |
| y-axis(fluorescent protein preservation ratio %) |  |  |  | 171.46 |
| y-axis(fluorescent protein preservation ratio %) |  |  |  | 171.61 |
| y-axis(fluorescent protein preservation ratio %) |  |  |  | 182.00 |
| y-axis(fluorescent protein preservation ratio %) |  |  |  | 182.93 |
| y-axis(fluorescent protein preservation ratio %) |  |  |  | 184.68 |
| y-axis(fluorescent protein preservation ratio %) |  |  |  | 185.12 |
